# Horticultural additives influence peat biogeochemistry and increase short-term CO_2_ production from peat

**DOI:** 10.1007/s11104-024-06685-9

**Published:** 2024-05-09

**Authors:** Bidhya Sharma, Tim R. Moore, Klaus-Holger Knorr, Henning Teickner, Peter M. J. Douglas, Nigel T. Roulet

**Affiliations:** 1 Department of Geography, McGill University, Montreal, Canada; 2 Institute of Landscape Ecology, University of Münster, Münster, Germany; 3 Department of Earth and Planetary Sciences, Geotop Research Center, McGill University, Montreal, Canada

**Keywords:** Horticultural peat, Growing media, Horticulture, Peat decomposition, CO_2_ fluxes, Peat biogeochemistry

## Abstract

**Aims:**

Peat is used as a major ingredient of growing media in horticulture. Peat extracted from bogs can be acidic and low in nutrient availability and is therefore mixed with liming agents, nutrients, surfactants, perlite and so on. This study aims to estimate the rates at which raw peat and the modified peat (‘growing media’) decompose to release carbon dioxide (CO_2_), to estimate the release of carbon (C) from liming agents and to estimate how peat biogeochemistry is changed.

**Methods:**

We obtained 28 and 24 samples of raw peat and 24 growing media from four peat extraction companies in Canada. Growing media were treated with horticultural additives. We incubated the samples under laboratory conditions, measuring CO_2_ production, tracer using ${\updelta }^{13}{\text{C}}$-${{\text{CO}}}_{2}$, pH, C, nitrogen (N) content and humification indices (HIs) from infrared technology called Fourier transform-mid infrared (FT-MIR).

**Results:**

C:N ratio, pH, dissolved organic carbon, bulk density and C content differed significantly (*P* < 0.05) between raw peats and growing media. There was more than a doubling of total ${{\text{CO}}}_{2}$ production from growing media compared to raw peat. HIs show higher values for the growing media, which could result from spectral band shifts in the growing media because of increased cation availability. ${\updelta }^{13}{\text{C}}$-${{\text{CO}}}_{2}$ as a tracer showed an average 22% of the total ${{\text{CO}}}_{2}$ production orginated from added carbonate materials.

**Conclusion:**

Our results provide the rates (0.15 ± 0.017mgCO_2_-Cg^−1^d^−1^) at which horticultural peat decomposes and on the source of emitted ${{\text{CO}}}_{2}$. This will improve current estimates CO_2_ emissions from horticultural peat.

**Supplementary Information:**

The online version contains supplementary material available at 10.1007/s11104-024-06685-9.

## Introduction

Peatlands are prominent features of the Canadian landscape, storing about 147 Gt Carbon (C) (Tarnocai et al. 2002). Of the estimated 1.14 million km^2^ of peatlands in Canada (Xu et al. 2018), around 0.03% are being actively extracted for use in horticulture (CSPMA 2017)with an annual average dry peat extraction of 0.9 Mt yr^−1^ between 2015 to 2022 (Natural Resource Canada 2022). Assuming 50% of the peat extracted is C, 0.45Mt ${\text{C}}$ are removed from Canadian peatlands each year. As not all the C extracted is emitted back to the atmosphere, what happens to this irrecoverable C remains an important question to accurately account the C lost from horticulture peat use. Compared to other human disturbances in Canadian peatlands, peat extraction for horticulture is one of the smallest disturbances in terms of area coverage in Canada (Harris et al. 2021). Extracted horticultural peat is used by professional and amateur growers for food production, ornamental plants, gardening, landscaping and mineral soil improvement, among other purposes. The use of peat is common and in demand in horticulture, and is in increasing demand due to its well-known and favourable properties and a current lack of viable alternatives (Alvarez et al. 2018).

In their C accounting, the International Panel on Climate Change assume all the peat C harvested to be instantaneously released back to the atmosphere (Eggleston et al. 2006). While the rapid loss of peat C might be accurate if the peat is used as fuel, peat decomposition follows an exponential decay, with the C released slowly over time. A significant fraction of greenhouse gas (GHG) emissions from peat extraction is due to peat decomposition over time (Cleary et al. 2005). How much of the extracted C is emitted to the atmosphere and in what time frame becomes important, allowing accurate reporting of emissions from peat use for the horticultural sector. This could allow to quantify if subsequent accumulation of C in restored peatlands compensates for the loss of extracted peat (Nugent et al. 2019) and it permits comparison of the C footprint of peat with that of alternative growing media like coconut coir and wood fiber.

In the extraction process, peatlands are drained, and the vegetation is removed. The aerobic conditions created by the process accelerate heterotrophic respiration compared to anaerobic conditions (Laiho 2006). Peat is then extracted using vacuum harvesters and stored in stockpiles in the extraction fields. As peat extracted from ombrotrophic bogs can be acidic and low in available nutrients, several nutrients, horticultural additives can be mixed to optimize its physical and biogeochemical properties for plant growth in horticulture. After mixing with additives, peat is called a growing media. Additives may be limestone/dolomite, inorganic fertilizers, perlite and surfactants. Once the additives are mixed in, the growing media are often bagged and shipped to professional and amateur growers to use in horticulture. As conditions are made optimal for plant growth, the rate of decomposition is potentially altered compared to raw peat. Several studies have shown that the decomposition rate of peat varies with both intrinsic biogeochemical properties (e.g. pH, nutrient availability and organic matter ‘quality’) as well as extrinsic factors (e.g. temperature and particularly degree of saturation) (Andersson & Nilsson 2001; Blodau et al. 2004). As a result of aerobic conditions, raised pH, and improved nutrient availability, higher microbial activity and decomposition rates are expected in growing media than in raw peat. Consequently, the faster cycling of C in growing media would potentially translate into more decomposition, because of the stimulating effect of horticultural additives on microbial activity (Pot et al. 2022).

Several studies have measured biogeochemical properties and/or decomposition rates of peat with one or more components of horticultural additives (Andersson & Nilsson 2001; Li et al. 2022; Pinsonneault et al. 2016). However, the set of horticultural additives differs among companies and for products within the same company. Studies available on growing media often focus on how media quality improves plant growth and usually analyze only a single type of growing media sourced from one company (Leiber-Sauheitl et al. 2021; Lévesque et al. 2018). Therefore, a more comprehensive understanding of growing media properties and decomposition rate remains desirable for use in C accounting.

The primary objective of this study is to measure emissions from growing media and compare them to emissions from raw peat. The ‘recipes’ to make growing media are numerous and vary among and even within the companies depending on the targeted use of the product. Therefore, to put the measurements of emissions in context, we investigated if the emissions can be explained by the measured biogeochemical properties.

The growing media pH is raised using calcitic $\left[{{\text{CaCO}}}_{3}\right]$ or dolomitic $\left[{\text{CaMg}}{\left({{\text{CO}}}_{3}\right)}_{2}\right]$ limestone, which when dissolved in acidic water, releases carbon dioxide (CO_2_). In previous studies with limed soils, direct CO_2_ emissions from the lime are persistent and remain over a long period (Biasi et al. 2008). Liming in agricultural soil is a common practice in acidic soils, and lime-based national emissions are accounted for in agricultural emissions in Canada (Environment & Climate Change Canada 2023). Therefore, partitioning the emitted CO_2_ into biotic (peat-based) and limestone sources needs to be addressed for accurate measurement and reporting of biotic emissions from growing media. The stable isotopic composition of CO_2_ can be used to separate the ${{\text{CO}}}_{2}$ flux into abiotic and biotic components owing to the different ^13^C abundance in peat and limestone, using a two-way mixing model (Fry 2006).

Recently, FT-MIR derived humification indices (HIs) have been widely used to characterize peat properties and have been linked to several decomposition proxies (Broder et al. 2012; Drollinger et al. 2020; Harris et al. 2023). Previous studies have used FT-MIR results to detect short-term changes in the chemical properties of peat following incubation (Tfaily et al. 2014). Given the ease and low cost of FT-MIR analysis, we wanted to explore the usability of HIs in the horticultural peat context to assess peat decomposition.

Previous attempts to model climate impact of peatland restoration on peat extraction sites excluded the C removed from the systems (Nugent et al. 2019). Yet, removal of peat C and its decomposition in ex situ environments are previously reported to be the largest source of GHG emissions for the Canadian peat industry (Cleary et al. 2005). Our measurements of CO_2_ emissions from decomposing growing media allow more accurate upscaling and budgeting of CO_2_ emissions from Canadian horticultural peat extraction.

## Materials and methods

### Sample collection and preparation

In July 2020, we contacted four peat harvesting companies based in Quebec and Alberta and requested samples of raw peat from harvesting sites and growing media (horticultural additives added to the raw peat) ready for sale. We asked for variations in peat and growing media and received 28 peat samples and 24 growing media samples. Each company had its definition of ‘peat quality’, so we reclassified the grade groups based on the von Post scale that ranged in our case from 3 to 8 (Table 1) (Rydin and Jeglum 2005). Samples were divided into triplicates and stored at 4°C before laboratory analysis. Measurements of incubation for CO_2_ and δ^13^C- CO_2_ were done on triplicate sub-samples, whereas all other analyses were done on a representative single sub-sample only.

**Table 1:** Samples divied into groups using type (peat or growing media) and a measure of peat quality (using von Post groups). Sample size is the number of samples in that particular sub-group

Type	Quality	Sample size
Peat	von Post 3–4	*n* = *10*
	von Post 5–6	*n* = *9*
	von Post 7–8	*n* = *9*
Growing media	von Post 3–4	*n* = *12*
	von Post 5–6	*n* = *6*
	von Post 7–8	*n* = *6*

### Laboratory analysis

Gravimetric moisture content was based on mass loss from 10 g of fresh peat samples upon oven drying at 105 °C for 24 h. Loss-on-ignition (LOI) was determined using 1- 2 g of oven-dried samples ignited at 550 °C for 4 h (Heiri et al. 2001). Our measurement of LOI represents organic matter content. A higher temperature of combustion is needed to combust inorganic compounds (Heiri et al. 2001). In hindsight it would have been useful to obtain a measure of inorganic carbon content, but we did not. The peat pH was measured in water with 1:35 dry mass to water mass ratio (Nilsson et al. 1995). Bulk density was calculated as ratio of dry mass of a known volume of 50 cm^3^ peat that was obtained in peat bags. Samples were oven-dried at 60 °C for 120 h and ground to a fine powder for total C, total N, and Fourier transform mid-infrared (FT-MIR) spectroscopy. For the analyses of C, N, solid δ^13^C, FT-MIR, to remove added carbonates finely ground samples were treated in 1 M HCl, left in the oven to evaporate and treated with deionized water until the pH of the peat and water solution was circum-neutral (raw peat samples were not treated with HCl). We evaporated the excess acid and DI water, instead of decanting excess solutions, to ensure that the soluble fractions of C are not poured off (Hélie 2009). C and N were measured using direct combustion (900 ^∘^C) with an elemental analyzer (Flash EA 1112 CN ThermoFinnigan, Waltham, MA, USA). We performed the isotopic measurements on solid peat with a Micromass model Isoprime 100 isotope ratio mass spectrometer coupled to an Elementar Vario MicroCube elemental analyser in continuous flow mode (GEOTOP, Montreal). Two internal reference materials (δ^13^C = -28.74 ± 0.02‰ & -11.80 ± 0.03‰) were used to normalize the results on the NBS19-LSVEC scale. A third reference material (δ^13^C = -17.06 ± 0.02‰) was analyzed as an unknown to assess the exactness of the normalization. Results are given in delta units (δ) in ‰ vs Vienna Pee Dee Belemnite (VPDB). The overall analytical uncertainty (1σ) was better than ± 0.1‰.

For FT-MIR, 2 mg of powdered sample was mixed with 200 mg KBr (FTIR grade, Sigma Aldrich, St. Louis, MO, USA), and spectra were obtained using a Cary 660 FTIR spectrometer (Agilent, Santa Clara, CA, USA). With a resolution of 2 ${{\text{cm}}}^{-1},$ spectra were recorded from $600\mathrm{ c}{{\text{m}}}^{-1}$ to $4000\mathrm{ c}{{\text{m}}}^{-1}$ and then baseline corrected (Beleites and Sergo 2021) and normalized with the irpeat package (Teickner and Hodgkins 2020) to estimate the relative heights of specific peaks. Humification indices (Broder et al. 2012) were computed to analyze relative abundances of groups of molecular structures relative to the absorption band at 1090 cm^−1^ (assumed to be caused predominantly by polysaccharides in this case because of low mineral contents):

- 1420/1090: phenolic and aliphatic structures / polysaccharides
- 1510/1090: aromatic $C=C$ or $C=O$ of amides / polysaccharides
- 1630/1090: aromatic $C=C$ and $CO{O}^{-},\mathrm{ protein}$ NH_2_ and C = O /polysaccharides
- 1720/1090: carbonylic and carboxylic $C=O$/ polysaccharides

#### Incubation experiments

We incubated triplicates of peat and growing media samples (~ 10 g) in 250 mL Mason jars after removing large roots and twigs. After adjusting the water content to 60% volumetric moisture, samples were stored at 4°C for one week to avoid the initial disturbance and brought out at room temperature for 48 h. Incubations to determine ${{\text{CO}}}_{2}$ emissions were done aerobically at a temperature of 23°C. Since the jars were not completely closed during the settling down period for nine days (one week at 4 °C and two days at 23 °C days), we assume that aerobicity in the bottles was maintained during the sampling period. Gas samples (5 mL) were collected from each jar using stopcocks attached to rubber tubes in the jar lids, and before sample collection, the headspace air was mixed by flushing the syringes. After 48 h incubation at 23°C, gas samples were collected at 0, 6, 24 and 48 h and ${{\text{CO}}}_{2}$ concentrations were measured on a Shimazdu GC-2014 gas chromatograph equipped with a methanizer and flame ionization detector. N_2_ was the carrier gas, the SRI column temperature was 70°C and the flame ionization temperature detector (FID) was at 110°C. Three to five standards of 5000 ppm were run through the GC before, during and after the sampling period. Five mL of ambient air were added to the jars after each sampling, and rates of ${{\text{CO}}}_{2}$ production by samples were calculated from the rates of change in concentration within the headspace and corrected for the dilution because of the 5 mL ambient air addition. For quality control, only measurements with r^2^ > 0.8 were used. Less than 10% of the data were discarded after the control. Production rates were expressed per mass of organic C (org C) in the peat or growing media, as the samples had varying C content.

#### Separation of $CO_2$ sources based on stable isotopic composition

Sub-samples of four peat samples, one from each company, and of all growing media were incubated as above in triplicate to measure the ${\updelta }^{13}{\text{C}}$ (V-PDB) signature of ${{\text{CO}}}_{2}$ emissions. 25 mL of headspace gas was sampled at 0, 2, 4 and 6 h and $5\mathrm{ mL}$ were used to measure ${{\text{CO}}}_{2}$ concentration, as above, and $20\mathrm{ mL}$ was used to determine δ^13^C- CO_2_ in a G2201-i CRDS Isotopic Analyzer system (Picarro, Santa Clara, CA). After each sampling, 25 ml of ${{\text{CO}}}_{2}$-free gas was refilled in the Mason jars. During each sampling period, two replicate CO_2_ standards of 850 ppm and -28.5‰ VPDB and an ambient air sample were run through the instrument. Measurements on the standards had a standard error of < 0.4‰ throughout the sampling period. The Picarro instrument was calibrated prior to the measurement period with two additional isotopic standards (100% CO_2_) with δ ^13^C values of -15.6 and -43.2‰ VPDB (Stix et al. 2017).

The δ^13^C of emitted CO_2_ was calculated using Keeling plots (Keeling 1958). Intercepts of δ^13^C values of CO_2_ were accepted when the regression coefficient was > 0.90 and when the coefficient of variation was less than 10%. Around 10% of sub-samples had a regression coefficients of less than 0.90, for these samples only two replicates were used in calculations. In addition, 5% of the samples had coefficient of variation larger than 10% and were removed from subsequent analyses in order to achieve high confidence in measurements of δ^13^C values. Intercept values for each sample and standard errors calculated from the triplicates can be found in Table S1. These quality check controls are similar to other studies using Keeling plots (Biasi et al. 2008; Pataki et al. 2003; Soper et al. 2017).

The δ^13^C signature was used to divide the total ${{\text{CO}}}_{2}$ flux into lime- and peat-based sources for the growing media. From the horticultural peat extraction companies, we requested samples of their commercially used limestone products. We received seven different limestone and dolomite products in total. For lime δ^13^C signature measurement aliquots of typically 100–150 μg of powdered samples were analyzed on a Nu Instruments Perspective™ isotope ratio mass spectrometer equipped with a NuCarb™ online carbonate preparation device at the McGill University, Geotop Stable Isotope Laboratory. On this instrument, carbonate powders are reacted in orthophosphoric acid at 70ºC and analyzed via dual inlet following double distillation of the evolved CO_2_ gas. Based on regular analysis of an in-house standard (UQ6), reproducibility is better than 0.1‰. One sample was removed for large variability between replicates. Measured average δ^13^C value of lime was -0.03‰ (0.28‰) and individual δ^13^C value for solid peat in a two pool mixing model equation (Biasi et al. 2008; Estop‐Aragonés et al. 2022; Fry 2006; Wild et al. 2023).

$$f=\frac{\delta -{\delta }_{0}}{{\delta }_{1}-{\delta }_{0}}$$

Here, $f$ is the fractional contribution of lime to total flux, $\delta$ is the isotopic signature for CO_2_ emitted from growing media, ${\delta }_{0}$ is the isotopic signature of solid peat and ${\delta }_{1}$ is the isotopic signature of lime.

#### Dissolved organic carbon (DOC), total dissolved nitrogen (TDN) and phenolic concentration

After the incubation, two grams of sample were mixed with 20 mL of distilled water for 1 h at 200 rpm in a shaker. After filtration with 0.45 µm filter papers (Macherey–Nagel, Düren, Germany), concentrations of DOC and TDN were determined using a Shimadzu TOC-TN analyzer (Shimadzu Corp., Kyoto Japan). Because of significant differences in C content among samples, DOC and TDN are expressed per g solid org C.

For phenolic concentration we adopted the method from Alshehri et al. (2020). Briefly, 5 g of the incubated sample were mixed with 40 mL of DI water in 50 mL centrifuge tubes and thoroughly mixed by shaking for 24 h at a speed of 200 rpm. Afterwards, samples were centrifuged at 5000 rpm for 30 min on a Sorvall ST16R centrifuge (Thermo Fisher, Altricham, UK). The samples were then filtered through 0.45 $\mathrm{\mu m}$ Macherey–Nagel filter papers. In a separate 2 mL centrifuge tube, 1 mL of filterate was added, followed by 50 $\mathrm{\mu L}$ of Folin–Ciocalteau phenol reagent and 0.15 mL of Na_2_CO_3_ (200 g L^−1^) to buffer the reaction. A range of standards of phenol compounds between 0.5 to 30 mg L^−1^ was prepared in a similar way. After 1.5 h, 300 $\mathrm{\mu L}$ of each sample and the standard were transferred to wells of a clear 96-well microplate. Absorbance was measured at 750 nm on an Epoch Microplate Spectrophotometer (BioTek Instruments Inc., Winooski, Vermont) and converted the values into phenol concentration per g org C.

### Statistical analysis

Peat with horticulture additives in them are growing media, presumably differing depending on the specific additions. We lack information required to match each growing media sample with the respective original peat, we treat peat and growing media as independent groups. Furthermore, we make the assumption that the differences we observe are due to horticultural additives, even though differences in the peat material can also contribute to some of the differences. The statistical analyses were conducted in R, version 4.1.0 (R Development Core Team 2021). We first discuss the differences between peat and growing media for each variable and then compare the results with the degree of decomposition for peat and growing media individually. Finally, we highlight the difference between peat and growing media within each von Post class. Both the independent variables, peat or the growing media and the von Post groups are treated as categorical variables and the interaction between the two variables is also considered.

We used the generalized least squares (gls) model in R package “nlme” for statistical comparison between the groups (Pinheiro 2009). Whenever the residuals of the models demonstrated heteroskedasticity, we used the varIdent variance structure in the gls model as it handles differences in variances of different groups (Supplementary information Section A). The choice between the model with equal and unequal variances was guided by a likehood-ratio test, comparing the models. Results from the models where residuals demonstrate homoscedasticity and higher log-likelihood values are reported. Post hoc comparisons among the groups were made using the package “emmeans”, which used the Tukey method to adjust for multiple comparisons. Unless otherwise stated, 10% is used as the significance level. For comparing δ^13^C- CO_2_ between peat and growing media, we used two sample t-test with unequal variance. We report Spearman correlation coefficients to estimate correlations between the variables, Correlations coefficients for significant relationships are termed moderate when r is between $\pm$ 0.3 to $\pm$ 0.5) or strong when |r| $>0.5$. Results are presented as the average $\pm$ one standard error.

## Results

We first describe the pooled differences between peat and growing media for biogeochemical properties and CO_2_ emissions. As the measured variables in each group (peat and growing media) also differ by the degree of decomposition, we present the results along the von Post gradient within each group. Finally, we report the differences in biogeochemical properties and overall and peat specific C emissions between the two groups (peat and growing media) within each von Post class.

### Biogeochemical properties

There were differences in biogeochemical properties between peat and growing media in their average pH, bulk density, water-soluble phenolic concentration and LOI (Table 2, Figure S1). The peat samples were more acidic than the growing media, with mean pH values of 4.16 $(\pm 0.12)$ and 5.78 $(\pm 0.16)$ respectively. Within peat, the pH of von Post class 7–8 was highest followed by class 5–6 and 3–4 respectively. This trend was not present for the growing media. When compared between peat and growing media in each von Post class, growing media always had a higher pH. On average, the growing media also had a higher bulk density than peat (0.09 $\pm 0.007$ and 0.07 $\pm 0.003$ g cm^−3^ for growing media and peat respectively). This difference in average appeared to be driven by growing media von Post class 7–8 which had the largest bulk density of all the groups.

**Table 2:** Biogeochemical properties mean ($\pm$ se) of peat and growing media in each von post groups. *n* = 28 for peat and *n* = 24 for growing media

Group	Von Post	pH	LOI (mass-%)	Bulk density (g cm^−3^)	Phenolic (mg g^−1^ org C)	Carbon (mass-%)	Nitrogen (mass-%)	C:N (g g^−1^)	δ^13^ *C* − *solid* (‰)
Peat	3–4	3.83 (0.01)	96.87 (0.09)	0.07 (0.001)	0.54 (0.02)	52.08 (0.15)	0.86 (0.01)	62.57 (0.93)	-27.00 (0.02)
	5–6	3.86 (0.02)	95.84 (0.41)	0.067 (0.001)	0.73 (0.04)	51.22 (0.28)	1.09 (0.02)	47.88 (0.93)	-26.65 (0.03)
	7–8	5.1 (0.10)	86.63 (0.93)	0.009 (0.002)	0.48 (0.04)	48.39 (0.43)	1.60 (0.04)	30.83 (0.73)	-26.91 (0.09)
Growing media	3–4	5.78 (0.09)	72.36 (1.26)	0.074 (0.001)	0.84 (0.03)	41.88 (0.72)	0.94 (0.02)	45.00 (1.06)	-27.16 (0.04)
	5–6	5.33 (0.09)	89.46 (0.67)	0.085 (0.004)	0.62 (0.03)	45.56 (0.50)	1.09 (0.03)	42.92 (0.88)	-27.27 (0.04)
	7–8	6.21 (0.10)	79.00 (0.81)	0.12 (0.004)	0.37 (0.02)	44.45 (0.73)	1.49 (0.02)	30.44 (0.91)	-27.57 (0.04)
Group	Von post	DOC (mg g^−1^org C)	TDN (mg g^−1^org C)	DOC: TDN	Hi1	Hi2	Hi3	Hi4	
Peat	3–4	1.25 (0.01)	0.09 (0.003)	16.02 (0.52)	0.51 (0.003)	0.33 (0.004)	0.67 (0.005)	0.61 (0.005)	
	5–6	1.21 (0.04)	0.08 (0.003)	14.95 (0.37)	0.60 (0.006)	0.46 (0.008)	0.84 (0.012)	0.71 (0.005)	
	7–8	1.04 (0.07)	0.12 (0.003)	8.30 (0.54)	0.91 (0.02)	0.84 (0.02)	1.30 (0.02)	0.80 (0.01)	
Growing media	3–4	2.13 (0.10)	0.48 (0.06)	8.09 (0.68)	0.57 (0.008)	0.39 (0.008)	0.76 (0.01)	0.41(0.01)	
	5–6	1.44 (0.05)	0.41 (0.03)	4.79 (0.40)	0.70 (0.009)	0.52 (0.01)	0.99 (0.01)	0.64 (0.004)	
	7–8	0.96 (0.05)	0.55 (0.03)	2.33 (0.17)	1.02 (0.02)	0.89 (0.02)	1.42 (0.02)	0.76 (0.009)	

Water soluble phenolic concentration was on average higher for growing media than for peat (0.58 $\pm 0.56$ and 0.61 $\pm 0.06\mathrm{ mg\;}{{\text{g}}}^{-1}\mathrm{\;org\;C}$ respectively). Peat samples did not demonstrate any observable patterns along the von Post scale, whereas for growing media, there was a decrease in phenolic concentration with increasing von Post class (Table 2, Fugure S1). LOI was significantly lower and more variable for growing media (80.3 $\pm 2.08\mathrm{ \%})$ than for peat (94.02 $\pm 1.06\mathrm{ \%},$ *P* < 0.001). LOI tended to decrease with an increase in von Post class, with LOI in peat for von Post class 7–8 being significantly different from classes 3–4 and 5–6 (*P* = 0.04 and *P* = 0.06 respectively). There was no observable pattern in LOI of the growing media along the von Post scale. Growing media LOI was lower than for peat in each von Post class.

Similar to LOI, there was an overall significantly higher organic C content (%) in the peat samples than in the growing media (means of 50.9 $\pm 0.51$ and 43.96 $\pm$ 1.07%, respectively) (Table 2, Figure S2). In contrast to the C content, N concentrations did not differ significantly between the two groups (1.11 $\pm 0.06$ for peat and 1.17 $\pm 0.06\mathrm{ \%}$ for growing media) or between each von Post group (Table 2, Figure S2). However, within peat, N concentrations were larger for more decomposed samples than for less decomposed samples. Similarly, growing media also had a larger N content for more larger von Post classes, with group 7–8 having the highest average LOI. The differences in C and N contents translated into a higher average C:N ratio for peat (50.44 $\pm 3.04)$ than the growing media (39.45 $\pm 2.01,$ *P* = 0.001). As expected, a decrease in C:N along the decomposition gradient was observed for peat as the C:N for von Post classes 3–4, 5–6 and 7–8 averaged 62.6 $\pm 2.44,$ 47.8 $\pm 3.11$ and 30.8 $\pm 3.33,$ respectively, and all the groups were statistically significantly different from one another (*P* = 0.02 between 3–4 and 5–6; *P* < 0.001 between 3–4 and 7–8 and *P* < 0.001 between 5–6 and 7–8 group) (Table 2, Figure S2). This gradient was less pronounced for growing media as only the von Post class 7–8 was statistically different from the other von Post classes. The average ${\delta }^{13}$ C-solid for peat samples was lower than for growing media (-26.88 and -27.37 ‰, respectively, *P* < 0.001) (Table 2, Figure S2). Along the von Post scale, there were no trends in ${\delta }^{13}$ C-solid for peat samples, whereas for growing media, average ${\delta }^{13}$ C-solid decreased with larger von Post class, but no statistical difference was observed ( P > 0.5 between all von Post group comparisons). Contrasting peat and growing media among each von Post class, statistically significant differences are observed for von Post class 5–6 (*P* = 0.005, difference of 0.7 ‰).

**Fig. 1:**
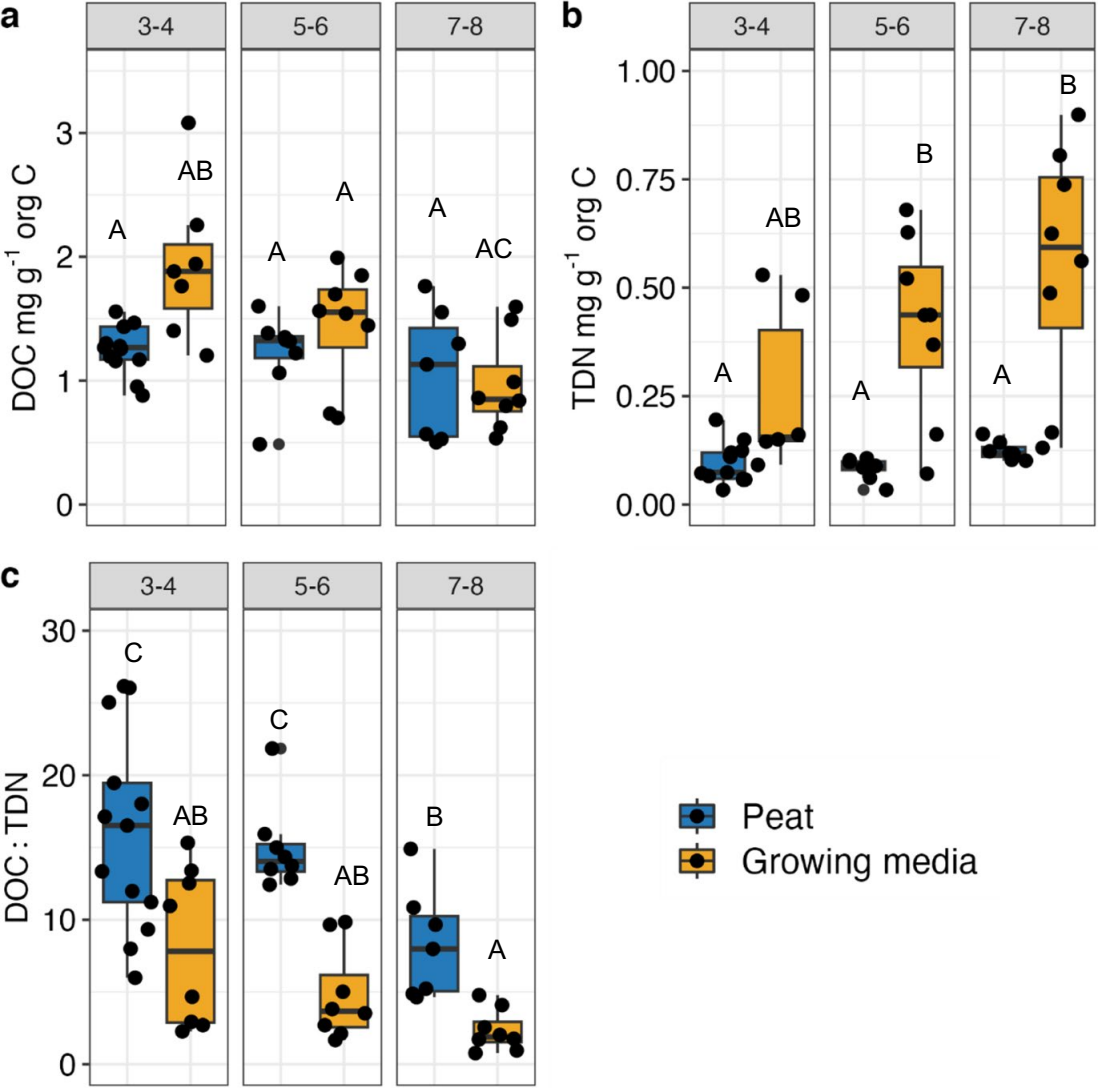
Values of **a**) DOC, **b**) TDN and **c**) DOC:TDN are shown for peat and growing media across different von-post class. Letters above each box represent significant difference as compared to other groups, where differing letters denote statistical difference

Average DOC in the growing media was higher than for peat (0.64 $\pm$ 0.04 and 0.5± $0.04 {\mathrm{mg\;g}}^{-1}\mathrm{\;org \;C},$ respectively, *P* = 0.02) (Table 2, Fig. 1). For both peat and growing media, DOC decreased with increasing von Post class. On the other hand, TDN was, on average, 3 to 5 times larger in the incubations of growing media than in the incubations of peat (0.484 $\pm 0.06\mathrm{\;and\;}0.10 \pm 0.005\;{\mathrm{mg\;g}}^{-1}\mathrm{org\;C\;respectively}$, *P* < 0.001) (Table 2, Fig. 1). Similar to the overall difference between peat and growing media this relationship held for each of von Post class 5–6 and 7–8 (*P* = 0.04 and *P* = 0.02, respectively). These differences in TDN also resulted in a large difference in average DOC:TDN values between peat and growing media (5.08 $\pm 0.76\mathrm{\;and}$ 13.09 $\pm$ 0.86, respectively, *P* < 0.001) (Table 2, Fig. 1). This ratio tended to decrease along increasing decomposition for peat, with class 7–8 having the on average lowest values compared to class 3–4 (P = 0.04) and group 5–6 (*P* = 0.02). This trend along von Post scale was also similar for growing media, but the differences were not statiscally different (all *P* > 0.14). Consistent with overall differences, DOC:TDN for peat and growing media differed statistically in each von post class (*P* = 0.08, *P* < 0.001 and *P* = 0.03 for 3–4, 5–6 and 7–8 groups).

**Fig. 2:**
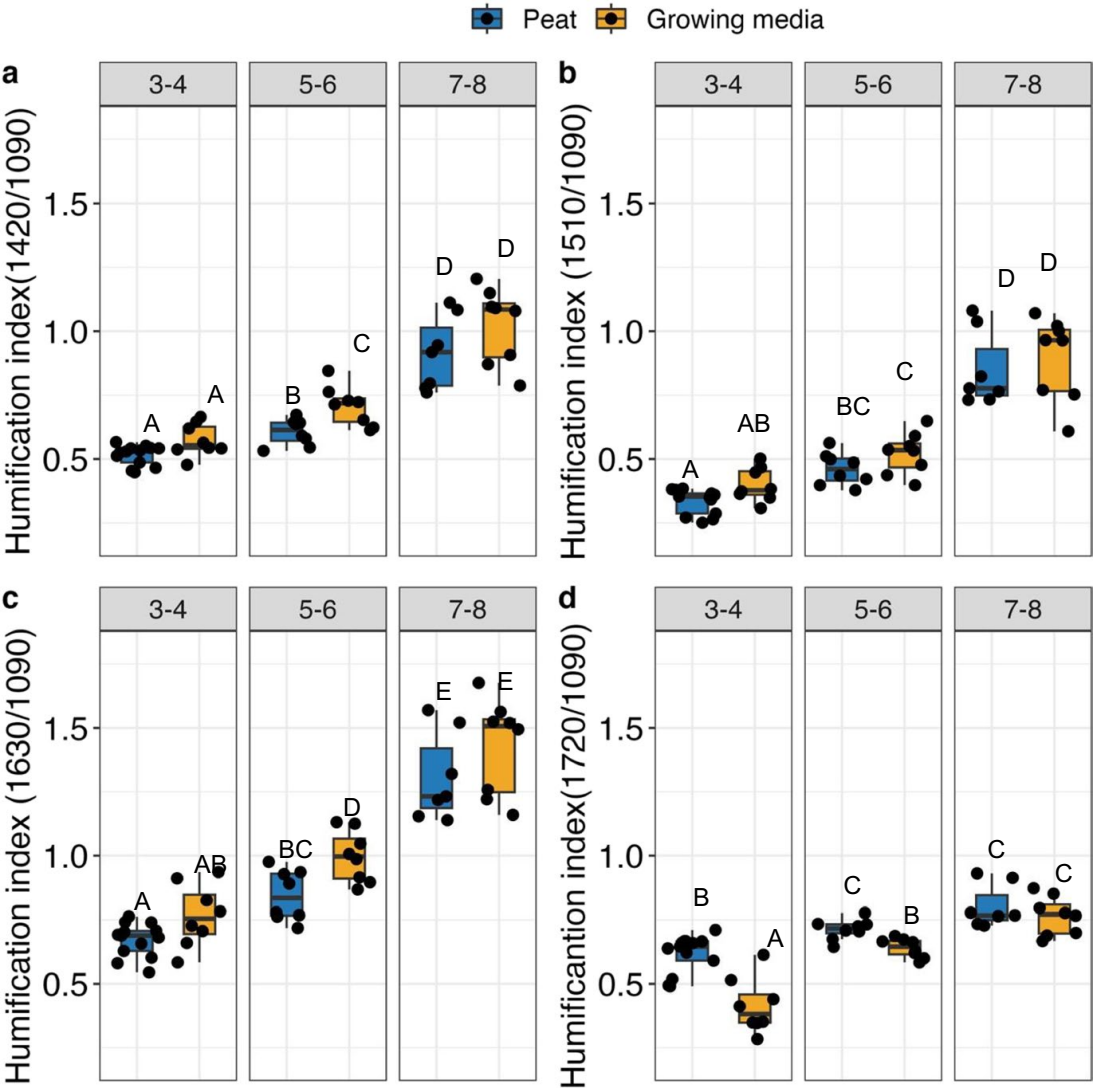
Humification indices **a**) 1420/1090 **b**) 1510/1090 **c**) 1630/1090 and **d**) 1720/1090 between peat and growing media across different von Post classes.The ratios are referred as Hi_1,_ Hi_2,_ Hi_3_ and Hi_4_ respectively. Letters above each box represent significant difference as compared to other groups, where differing letters denote statistical difference

In general, humification indices derived from FT-MIR differed along the von Post scale and between peat and growing media in each von Post class (Table 2, Fig. 2). For Hi_1_, Hi_2_ and Hi_3_, average growing media values always were larger values than for peat (*P* = 0.005; *P* = 0.08 and *P* = 0.003 respectively). However, Hi_4_ was on average smaller for growing media than for peat (*P* < 0.001). Humification indices Hi_1_, Hi_2_ and Hi_3_ differed along the von Post scale for both peat and growing media. For Hi_1_ and Hi_3_, only peat and growing media in class 5–6 differed (*P* = 0.07 for both), and for Hi_2_, none of the groups differed significantly (all *P* > 0.2). Average Hi_4_ of growing media in each von Post class always were smaller than for peat, and the differences were significant for class 3–4 (*P* = 0.005) and class 5–6 (*P* = 0.02).

### $C{O}_{2}$ emissions and δ ^13^C- CO_2_ measurements.

**Fig. 3:**
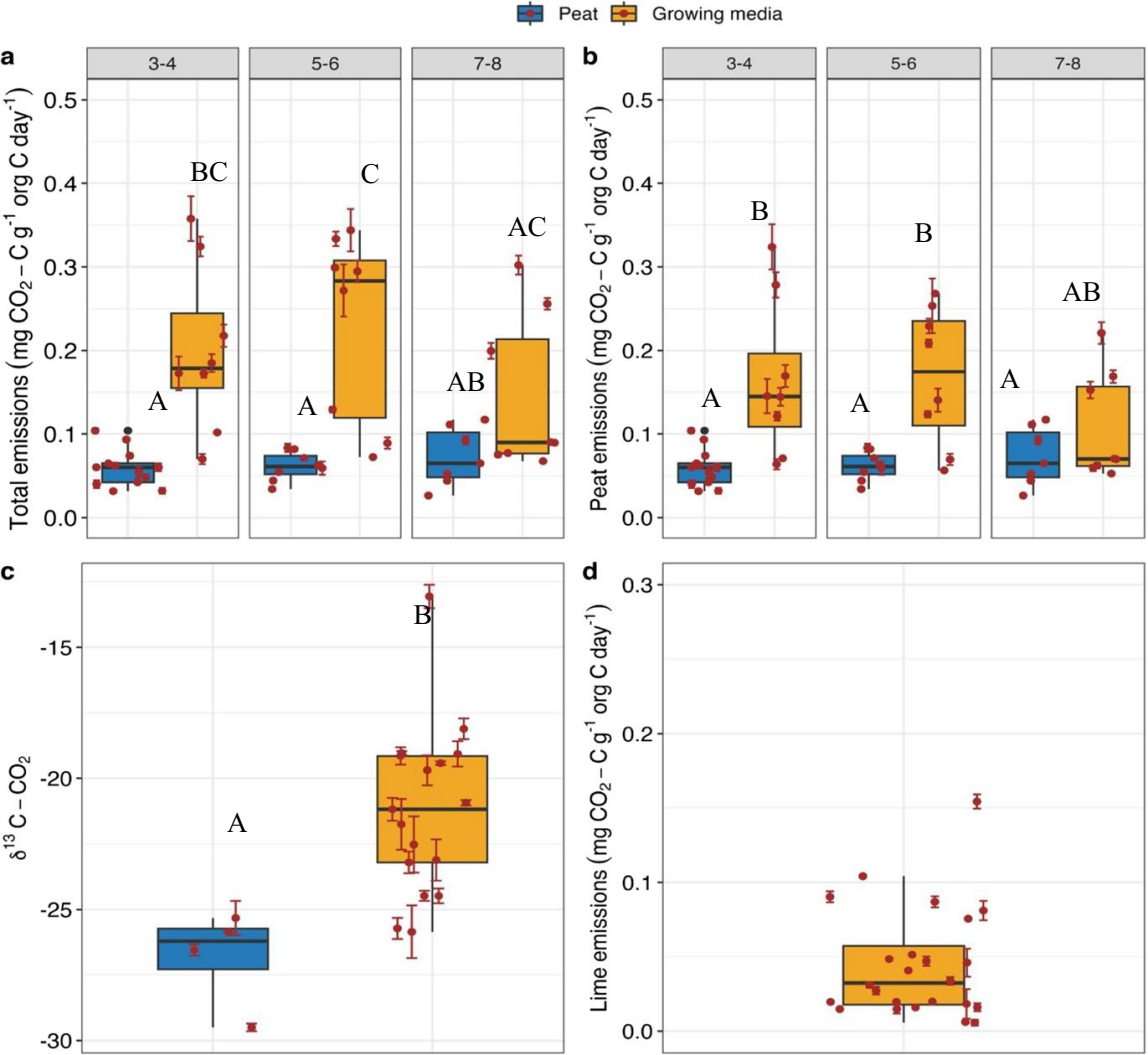
**a**) Total CO_2_ emissions, **b**) is the CO_2_ emissions after lime contribution has been removed for the growing media, **c**) δ ^13^C of the emitted CO_2_, and d) CO_2_ emissions from peat only. The bottom right graph in (**d**) emissions from lime in growing media. Numbers in the panel represent von Post classes. Differing letters above each box represent significant difference as compared to other groups

Total CO_2_ emitted from peat was on average three times larger for growing media than for raw peat (0.063 $\pm 0.004$ and 0.19 $\pm 0.02$ mg CO_2_-C g org C^−1^ day^−1^ respectively, t = 5.90, df = 23, *P* < 0.001). Variability in values, measured as the coefficient of variation of total emitted CO_2_, was larger for growing media than for peat (0.54 and 0.38, respectively). Neither for peat nor for growing media did the total ${{\text{CO}}}_{2}$ emissions differ statistically significantly along the von Post scale. Comparison within von Post classes showed larger and significantly different CO_2_ emissions for class 3–4 (t = 3.96, df = 7.42, *P* = 0.03) and class 5–6 (t = 4.15, df = 7.33, *P* = 0.03), whereas the difference was not significant for class 7–8 (t = 2.02, df = 9.12, *P* = 0.3) (Fig. 3a).

Average δ^13^C- CO_2_ values of peat were more negative than those of the growing media (mean of -26.80 and -21.22 ‰, respectively, *P *= 0.001), indicating the contribution of carbonates (relatively enriched in ^13^C) to the total emitted CO_2_ in growing media (Fig. 3b). The average fraction of carbonate emissions in the total flux from growing media was 22.3%, 0.05 mg CO_2_-C g org C ^−1^ day^−1^ (Fig. 3c). After subtracting the direct contribution of carbonates in growing media emissions (Fig. 3c and d), peat-based emissions in growing media were still larger than in peat (0.063 $\pm 0.004$ and 0.15 $\pm 0.017$ mg CO_2_-C g org C ^−1^ day^−1^ respectively, t = 4.62, df = 22.9, *P* < 0.001) (Fig. 3d). The peat-based CO_2_ emission did not differ significantly along the von Post scale for either peat or growing media. However, differences between peat and growing media in peat flux were significant, except for von Post class of 7–8 (*P* = 0.09, *P* = 0.05 & *P* = 0.74 for classes 3–4, 5–6 and 7–8 respectively).

### Correlation between variables

The combined correlation matrix and their associated *P-*values are shown in Table 3, and significant associations of CO_2_ emissions with explanatory variables are expanded in Figure S3. Most importantly, there was a moderate and significantly positive correlation of the peat-borne flux with pH (r_s_ = 0.41, P = 0.002), TDN (r_s_ = 0.55, *P* < 0.001) and DOC ( r_s_ = 0.39, *P* = 0.0013). Similarly, peat-borne C emissions show a moderate and negative association with C content in solid peat (r_s_ = -0.52, *P* < 0.001), with LOI (r_s_ = -0.49, *P* < 0.001), with DOC:TDN (r_s_ = -0.36, *P* < 0.007), and low and negative association with Hi4 (r_s_ = -0.34, *P* = 0.01).

**Table 3:** Correlation values following spearman rank correlation and associated p-values for assessing relationship between different measured variables. All the variables that are associated at 10% significance level are presented in bold

	Peat C emission	pH	LOI	Bulk density	DOC	TDN	DOC:TDN	C	N	C:N	Hi_1_	Hi_2_	Hi_3_	HI_4_	Phenol
pH	**0.38**														
	0.004														
LOI	**-0.47**	-**0.76**													
	< 0.001	< 0.001													
Bulk density	0.11	**0.46**	**-0.37**												
	0.41	< 0.001	0.006												
DOC	**0.43**	-0.13	-0.11	**-0.32**											
	0.001	0.3	0.41	0.01											
TDN	**0.46**	**0.65**	**-0.59**	**0.31**	0.15										
	< 0.001	< 0.001	< 0.001	0.04	0.26										
DOC:TDN	**-0.32**	**-0.68**	**0.52**	**-0.38**	0.22	**-0.88**									
	0.01	< 0.001	< 0.001	0.005	0.1	< 0.001									
C	**-0.52**	**-0.72**	**0.7**	**-0.28**	-0.04	**-0.63**	**0.57**								
	< 0.001	< 0.001	< 0.001	0.04	0.7	< 0.001	< 0.001								
N	-0.01	**0.38**	-0.2	**0.27**	**-0.48**	0.19	**-0.43**	-0.16							
	0.93	0.004	0.14	0.04	< 0.001	0.15	0.001	0.24							
C:N	-0.19	**-0.64**	**0.48**	**-0.33**	**0.37**	**-0.44**	**0.61**	**0.53**	**-0.89**						
	0.16	< 0.001	< 0.001	0.014	0.006	0.003	< 0.001	< 0.001	< 0.001						
Hi_1_	0.11	**0.57**	**-0.48**	**0.55**	**-0.35**	**0.31**	**-0.47**	**-0.4**	**0.65**	**-0.67**					
	0.42	< 0.001	< 0.001	< 0.001	0.009	0.02	< 0.001	0.002	< 0.001	< 0.001					
Hi_2_	0.10	**0.53**	**-0.43**	**0.51**	**-0.39**	**0.23**	**-0.42**	**-0.34**	**0.73**	**-0.7**	**0.97**				
	0.44	< 0.001	< 0.001	0.001	0.004	0.09	0.001	0.012	< 0.001	< 0.001	< 0.001				
Hi_3_	0.11	**0.56**	**-0.466**	**0.53**	**-0.37**	**0.38**	**-0.44**	**-0.4**	**0.66**	**-0.67**	**0.98**	**0.97**			
	0.42	< 0.001	< 0.001	< 0.001	0.006	0.04	0.001	0.003	< 0.001	< 0.001	< 0.001	< 0.001			
Hi_4_	**-0.25**	0.07	0.01	**0.5**	**-0.51**	-0.13	-0.07	0.07	**0.55**	**-0.38**	**0.69**	**0.71**	**0.71**		
	0.06	0.59	0.93	< 0.001	< 0.001	0.34	0.57	0.58	< 0.001	0.005	< 0.001	< 0.001	< 0.001		
Phenol	0.17	-0.11	-0.002	-0.16	**0.53**	**-0.28**	0.18	0.018	-0.22	0.18	**-0.26**	**-0.24**	**-0.28**	**-0.28**	
	0.22	0.42	0.98	0.23	< 0.001	0.04	0.19	0.89	0.1	0.19	0.06	0.07	0.04	0.04	
δ^13^ C- Peat	-0.09	**-0.5**	**0.42**	**-0.52**	0.2	**-0.38**	**0.48**	**0.35**	-0.06	0.2	**-0.29**	**-0.25**	**-0.25**	-0.08	0.08
	0.5	< 0.001	0.001	< 0.001	0.14	0.004	< 0.001	0.009	0.67	0.15	0.03	0.07	0.06	0.53	0.56

Hi_1_, Hi_2_ and Hi_3_ were associated positively and significantly with pH, bulk density, N, and negatively with C:N, DOC, TDN, LOI and weakly with phenolic concentration. While dividing the correlation matrix into two groups for peat and growing media, differing relationships were observed (Table S2 and S3). CO_2_ emissions for peat tended to increase with increasing δ^13^ C-Peat (r_s_ = 0.39, *P* = 0.03). For growing media, CO_2_ emission tended to increase with increasing DOC (r_s_ = 0.36, *P* = 0.08) and tended to decrease with increasing N content (r_s_ = -0.37, *P* = 0.02) and increasing C content (r_s_ = -0.37, *P* = 0.07).

## Discussion

### Biogeochemical differences between peat and growing media

Peat pH, LOI, C:N, phenolic content are within ranges and similar to the values reported for bog peat and for peat extracted for horticulture. For instance, from the data collected from undisturbed Ontario bogs the estimated 99% CI of i) pH ranged from 4.72 to 4.9, ii) LOI from 93.93 to 94.78% and iii) C:N from 32.62 to 35.56 (Riley 1994). The addition of horticultural additives affected several biogeochemical properties. Values of LOI, C:N, ${\updelta }^{13}{\text{C}}-{\text{C}},$ bulk density, phenolic concentration in a natural peatland are often used as a proxy for the decomposition stage; for example lower C:N signifies a more mineralized peat (Biester et al. 2014). However, most of these biogeochemical measures in growing media would be influenced by added inorganic fertilizers, lime and other inorganic buffers, therefore they would not be reflective of the degree of decomposition or biological origin of peat anymore (Fig. 2 and 3). The bulk density measurements on the compacted samples received in peat bags do not reflect bulk density as measured in natural peatlands. Although lower LOI in a natural peatland may suggest increased mineralization (Chambers et al. 2011) in our investigation, the lower LOI measured for growing media is influenced by inert perlite, confirmed visually and from the peat producers, and other potentially added inorganic substances. However, it remains unclear from this study whether the addition of perlite to peat directly impacts C mineralization. The addition of inorganic C, in the form of limestones, affected several key properties of growing media (e.g. pH, decomposition rate). Therefore, quantifying the amount of inorganic C added to growing media should be considered in future studies.

### CO_2_ emissions and influence of liming

CO_2_ emitted from raw peat (0.026 to 0.12 mg CO_2_-C g org C^−1^ d^−1^) measured in this study is on the lower end but within the ranges reported for other raw peat soils where total C is almost exclusively organic C. Glatzel et al. (2004) measured emissions from 0.027 to 0.7 mg CO_2-_C g g C^−1^ d^−1^ in a horticultural peat extraction site and a pristine bog. Similarly, Scanlon and Moore (2000) report emissions from 0.07 to 0.36 mg CO_2_-C g C^−1^ d^−1^ from a Canadian bog at 14 $\mathrm{^\circ{\rm C} }$. Potentially more similar conditions to our study are from Clark et al. (2023), where CO_2_ emissions from incubation of peat from actively extracted peatlands in Quebec ranged between 0.006 and 0.03 mg CO_2_-C g C^−1^ d^−1^, with C being predominantly organic.

Total CO_2_ emissions for growing media in our study (0.055 to 0.35 mg CO_2_-C g org C^−1^ d^−1^) are similar in magnitude with what has been reported for agricultural organic soils that are limed and fertilized in Finland with values ranging from 0.12 to 0.47 mg CO_2_-C g C^−1^ d^−1^ (Biasi et al. 2008). As we did not consider dissolved CO_2_ in water, considering that our setup volume was 250 mL and assuming a typical representative concentration of CO_2_ in the headspace in the observed ranges of pH, we underestimated CO_2_ production rates by a maximum of 20% depending on the exact pH (Stumm and Morgan 2012) for both peat and growing media.

Values of δ^13^C-CO_2_ for peat in our study (-24.66 to -26.9 $\permille$) are similar to values reported for unlimed plots in agricultural organic soils in Finland (-25.32 to -29.5 $\permille$) (Biasi et al. 2008). Values of δ^13^C- CO_2_ of growing media (-13.06 to -29.50 $\permille$) are also within the range reported for limed and fertilized plots by Biasi et al. (2008). The contribution of lime-derived CO_2_ to the total flux is on average 22.3%. Uncertainty in this measure could arise from the fractionation between solid peat and resulting CO_2_, or between the lime carbonate and the resulting CO_2._ A substantial fractionation between carbonate and the resulting CO_2_ has been inferred in soils at higher pH and with significant HCO_3_^−1^ leaching (Schindlbacher et al. 2015) but, we argue that at lower pH and with no HCO_3_^−1^ leaching in our closed incubation, fractionation of carbonate from dissolution and exsolution would likely be either neligible or similar to the fractionation that occurs in biotic respiration. However, even if the most extreme value of the fractionation value of 12‰ were to be considered in this study (Schindlbacher et al. 2015), on average it will alter the fractional contribution of carbonate from 0.22 to 0.39. In this scenario, the average biotic emission for growing media will decrease from 0.15 to 0.11 mg CO_2_-C g org C^−1^ d^−1^, but still validate our results that emissions for growing media almost twice as high as that for peat. Therefore, even while accounting for the uncertainities associated with lime-derived δ^13^CO_2_, we demonstrate that without partitioning the total flux into peat-based and lime-based, emissions from growing media would have been overestimated.

The measurements of at least twice as much biotic CO_2_ emissions for growing media compared to peat might be due to the indirect influence of additives that increased the pH and lowered the C:N ratio (Figures S1 and S2 and Table 3) and availability of DOC and TDN (Fig. 1 and Figure S3). These soil properties have been shown to impact microbial structure and activity, which in turn control the decomposition rate (Ren et al. 2018). For instance, limed-peat media had a different microbial community structure than unlimed-peat media (Pot et al. 2022) and increased C mineralization as a function of pH (Montagne et al. 2015). Thus, increase in pH following liming been shown to increase respiration rates and microbial activities in incubation samples where lime was applied in field conditions (Andersson and Nilsson 2001; Andersson et al. 2000). In addition, the direct contribution of added lime-derived CO_2_ has also been demonstrated even after several years of lime addition (Biasi et al. 2008). After portioning lime-derived CO_2_, the biotic emissions from growing media in our study (0.05 to 0.32 mg CO_2_-C g org C^−1^ d^−1^) fall into the range of what has been reported for disturbed agricultural peatlands (0.012 to 0.57 mg CO_2_-C g C^−1^ d^−1^ by Säurich et al. (2019)). Incubation at 20 $\mathrm{^\circ{\rm C} }$ of peat from a forest, cropland and grassland in Switzerland which has comparable pH, SOC and C:N ratio as to our study report an average emissions of 0.18 mg CO_2_-C g C^−1^ d^−1^ (Bader et al. 2018). Even though the biotic peat-based emissions are twice as large for growing media than for peat, current IPCC reporting (Eggleston et al. 2006) that 100% of peat extracted for horticulture is lost in a single year is over-estimated. For instance, an average 0.45 Mt C per year of peat is removed from Canadian peatlands (Natural Resource Canada 2022). Assuming a single average value (0.15 ± 0.017 mg CO_2_-C g org C ^−1^ day^−1^) for growing media decomposition; extrapolation from our results show that on the first year of extraction, a resulting amount of 0.024 Mt C (95% CI 0.019 to 0.03 Mt) is released back to the atmosphere as CO_2_ (Supplmentary information, Text C). In the 18,000 ha of extracted peatland harvesting sites in Canada that are under restoration (Environment and Climate Change Canada 2023), a long-term annual sink of 50 gC m^−2^ yr^−1^ following restoration (Nugent et al. 2019) means that only 0.009 Mt of C is sequestered into the restored peatlands. This amount of C sequestration that happens in currently restored peatlands is lower than what is emitted from peat extracted within a year of extraction (0.024 Mt C). In addition, if we consider the emissions from peat extracted over a longer timescale, the sequestration potential is small compared to the current level of extraction. However, the emissions that we report for growing media could differ once plants are introduced, in its use as spent-peat-based growing media (Vandecasteele et al. 2023) and compared to the after-use conditions to which the growing media is subjected. While the influence of plants could be important in shorter time-scales, the after use conditions to which peat is subjected to is important at a longer time-scale. Future work on these topics would be important to further constrain the IPCC reporting to adequately represent horticulture use of peat.

### Decomposition and humification indices

There are many different proxies for decomposition ranging from C:N, N, bulk density to $\delta$ ^13^C, MIR-derived humification indices and DOC in peat (Biester et al. 2014; Broder et al. 2012; Drollinger et al. 2020; Tfaily et al. 2014). For our original peat, our data similarly indicated that more decomposed peat has larger humification indices, smaller C:N, C and increased N, resulting in a decreased C:N ratio. In contrast, growing media samples did not show such trends (Figure S2 and S1). In addition, correlations between peat properties within peat samples (Table S3) indicate that larger humification index values relate to C:N, C, N and bulk density measurements. However, except for the positive relationship with TDN, none of the variables correlated with $\delta$ ^13^C values in peat samples. This could be because the range of $\delta$ ^13^C values in our study is quite narrow (1.4 ‰) and, in addition, our samples have peat that is sourced from different companies in different geographic locations. Different vegetation that contributed to the isotopic signature may have played a greater role in controlling $\delta$ ^13^C values in our case than decomposition processes (Hornibrook et al. 2000).

Humification indices derived from FT-MIR has been shown to be sensitive enough to detect small changes in peat chemistry that occur in just over 75 days of decomposition (Tfaily et al. 2014). However, larger values in growing media Hi_1_ $(1420/1090)$, Hi_2_(1510/1090) and Hi_3_ (1630/1090) in our study are potentially due to interactions of carboxyl groups with cations from the added lime (Ellerbrock and Gerke 2021) and not mainly due to decomposition. Interestingly, lower values of Hi_4_ (1720/1090) for growing media can also indicate the influence of added cations in the spectra (Ellerbrock and Gerke 2021): The band at 1720 cm^−1^ is caused, to a large fraction, by C = O stretching in carboxylic acids and increasing the pH value by adding lime will cause deprotonation of COOH groups and will cause cation exchange of protons for Ca^2+^, thus converting COOH groups into carboxylate COO^−^ groups with Ca^2+^ either bound electrostatically or as complex. This causes a decrease in absorption around 1720 cm^−1^ (Ellerbrock and Gerke 2021) and can explains lower Hi_4_ (1720/1090) in growing media than in peat. The same mechanism may have caused an increase in absorption around 1630 and 1420 cm^−1^, causing larger Hi_1_ (1420/1090) and Hi_2_(1630/1090) in growing media (Ellerbrock and Gerke 2021). Even if there are differences in the relative amounts of carbohydrates and aromatics, the influence of cations on carboxyl groups is a plausible confounder which will hamper the interpretation of humification indices in decomposition between peat and growing media. However, the patterns in Hi_1_ $(1420/1090)$, Hi_2_(1510/1090) and Hi_3_ (1630/1090) for both peat and growing media suggest that a rough overview of degree of decomposition can be obtained from FT-MIR analysis also for growing media, although changes over time in incubations are obscured.

## Conclusions

We characterized the biogeochemical properties of peat and compared them with growing media across their different grades. Horticultural additives of lime and inorganic fertilizers in the growing media caused marked differences in their pH, bulk density, C:N, DOC and TDN. Due to favorable changes in the environment for microbes from liming, addition of fertilizers and direct chemical dissolution of carbonate-based additives, we measured twice larger CO_2_ emissions from growing media than for peat Even after accounting for the direct CO_2_ emitted from chemical dissolution of carbonates (~ 22% of the total emission), the indirect effect of horticultural additives caused a doubling of the microbial respiration measured in growing media as compared to peat (0.063 ± 0.004 and 0.15 ± 0.017 mg CO_2_-C g org C ^−1^ day^−1^ respectively). This increased microbial respiration observed in growing media could be the result of the sub-optimal conditions of low pH, lack of N and other nutrients in raw peat where decomposition is impeded. Once, these conditions are altered in growing media, increase in CO_2_ production is thus expected. FT-MIR based humification indices could not be used to infer on preferential use and loss of different C fractions because of the influence of cations from the added lime on absorbance of molecular structures of the growing media samples. This means that humification indices cannot be directly used to identify difference in decomposition between peat and growing media. However, trends of indices along the von Post gradient for growing media suggest that they could be used to obtain a rough overview on the degree of decomposition of the parent material and its inherent decomposability. While the role of horticultural plants and after-use conditions remain to be assessed our initial extrapolation, assuming the decomposition rate is substrate invariable, suggest that of 0.45 Mt C extracted from Canadian peatlands, ~ 0.024 Mt C (95% CI 0.019 to 0.03 Mt) is released back to the atmosphere in the first year of extracted peat use.

## Supplementary Information

Below is the link to the electronic supplementary material.

Supplementary file1 (DOCX 1937 KB)

## Data Availability

The datasets generated during and/or analysed during the current study are available from the corresponding author on reasonable request.
