# Supplementary material for: Horticultural additives influence peat biogeochemistry and increase short-term CO2 production from peat
Source: Plant Soil. 2024 May 9;505(1-2):449–64. doi: 10.1007/s11104-024-06685-9 (PMC11680665; doi:10.1007/s11104-024-06685-9)
Supplement: Supplementary file 1 — Supplementary file1 (DOCX 1937 KB) [file 11104_2024_6685_MOESM1_ESM.docx]

**SUPPORTING INFORMATION for:**

**Horticultural additives influences peat biogeochemistry and increases short-term CO_2_ fluxes from peat**


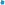


Bidhya Sharma^1^, Tim Moore^1^, Klaus-Holger Knorr^2^, Henning Teickner^2^, Peter M.J. Douglas^3^, Nigel Roulet^1^

^1^ Department of Geography, McGill University, Montreal, Canada

^2^Institute of Landscape Ecology, University of Münster, Münster, Germany

^3^ Department of Earth and Planetary Sciences, McGill University, Montreal, Canada

1. **Formulas used for model development.**

pH~ Amendment * von_post, weights = varIdent (form=~1|von_post* Amendment)

Bulk density ~ Amendment * von_post, weights = varIdent (form=~1|von_post* Amendment)

LOI ~ Amendment * von_post, weights = varIdent (form=~1|von_post* Amendment)

$\boldsymbol{B.}\boldsymbol{\delta}^{\mathbf{13}}\mathbf{C-C}\mathbf{O}_{\mathbf{2}}$ **Measurements**

Table S1: Average values ( $\pm\mathrm{SD})$of $\delta^{13}C-CO_{2}$ obtained after keeling plot method. Each value is an average of three replicates except for samples 27, 28,29, 51 and 52 for which only two replicates were used as regression coefficient were <0.9. Sample 44 is removed from subsequent analysis as the two sub-samples had regression coefficient less than <0.9.

| Sample | Type | $Intercept- \delta^{13}C$ (‰) | $SD$ (‰) |
| --- | --- | --- | --- |
| 1 | Peat | -26.54 | 0.37 |
| 5 | Peat | -29.5 | 0.24 |
| 13 | Peat | -25.86 | 0.01 |
| 37 | Peat | -25.32 | 1.12 |
| 2 | Growing media | -19.06 | 0.84 |
| 3 | Growing media | -24.47 | 0.33 |
| 4 | Growing media | -20.93 | 0.18 |
| 14 | Growing media | -24.48 | 0.48 |
| 15 | Growing media | -25.72 | 0.69 |
| 27 | Growing media | -23.11 | 1.36 |
| 28 | Growing media | -19.42 | 0.12 |
| 29 | Growing media | -22.52 | 1.85 |
| 30 | Growing media | -21.75 | 1.67 |
| 44 | Growing media | -7.44 | 1.10 |
| 45 | Growing media | -19.03 | 0.12 |
| 46 | Growing media | -19.14 | 0.58 |
| 47 | Growing media | -23.21 | 0.71 |
| 48 | Growing media | -13.06 | 0.76 |
| 49 | Growing media | -25.85 | 1.74 |
| 50 | Growing media | -18.10 | 0.68 |
| 51 | Growing media | -21.18 | 0.75 |
| 52 | Growing media | -19.69 | 1.00 |

1. **Extrapolation of CO_2_ emissions to Canadian scale**

Dry peat C extracted in Canada in 2023= 0.45Mt

Average CO_2_ emission calculated for growing substrate = 0.15 ± 0.017 mg CO_2_-C g org C ^-1^ day^-1^

Decomposition rate (k)value per year= 0.0547 (± 0.0062)

Extrapolated CO_2_ emissions for growing substrate in the first year of extraction = extracted amount * k value

= 0.024 Mt [95% CI= 0.019 to 0.03 Mt]


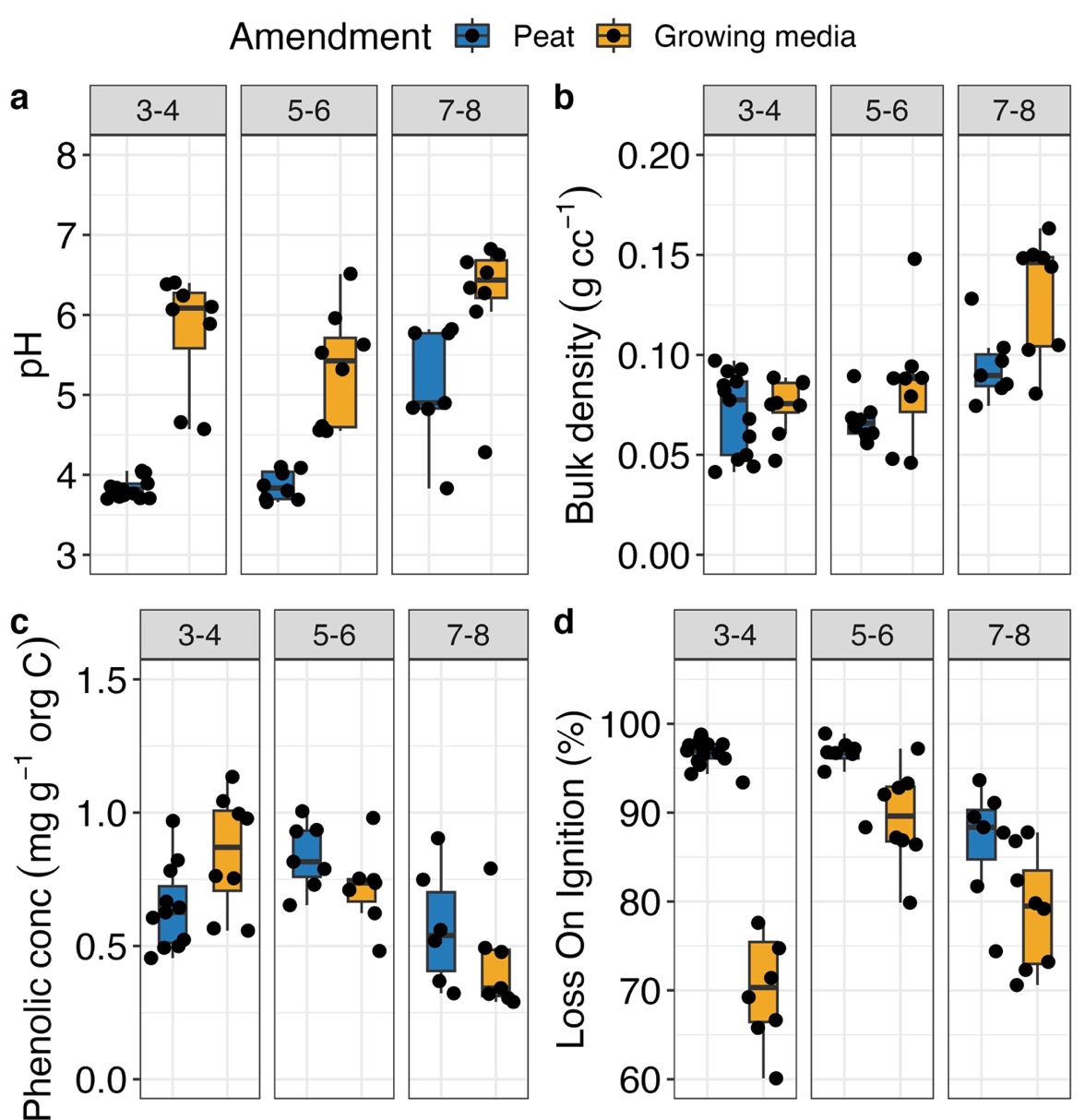


A

AB

AB

AB

AC
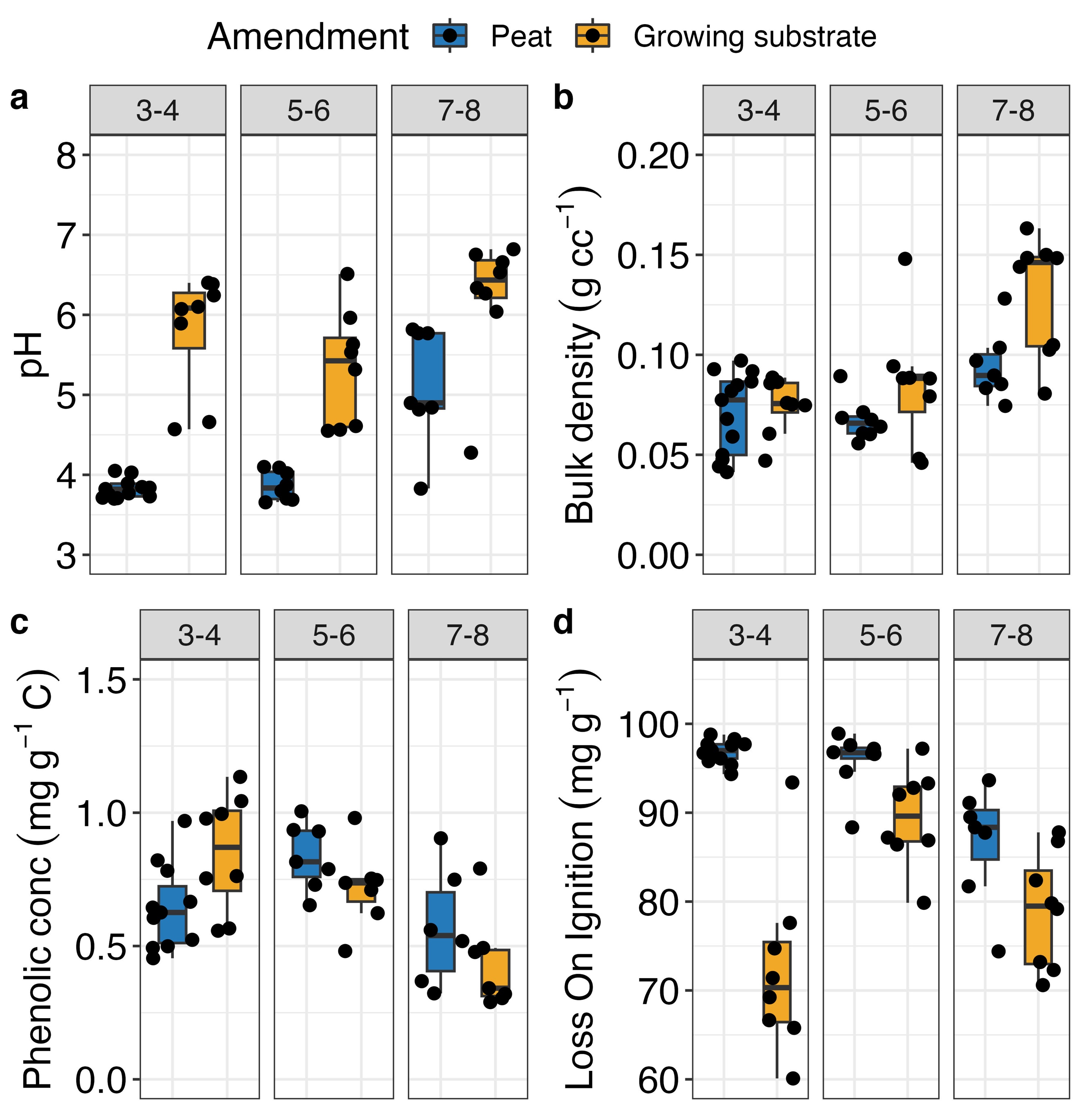


A

AB

A

A

A

AC

A

E

AB

BC

BD

DEF

A

B

A

B

B

B

C
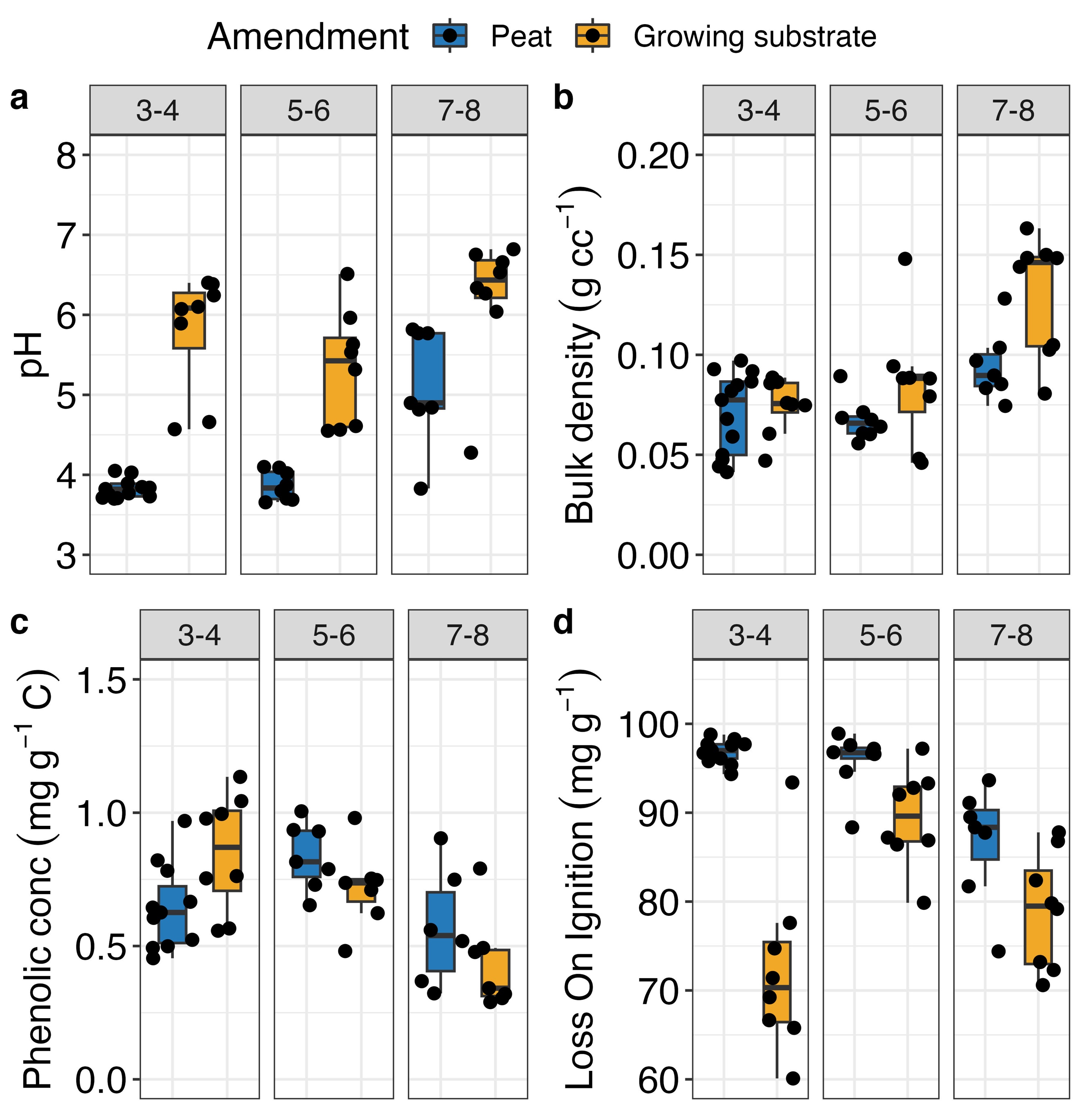


Figure S1: Biogeochemical properties a ) pH, b) bulk density (in the bags the samples were shipped in) c) phenolic concentration and d) LOI of peat and growing media classified across different von Post scale values.


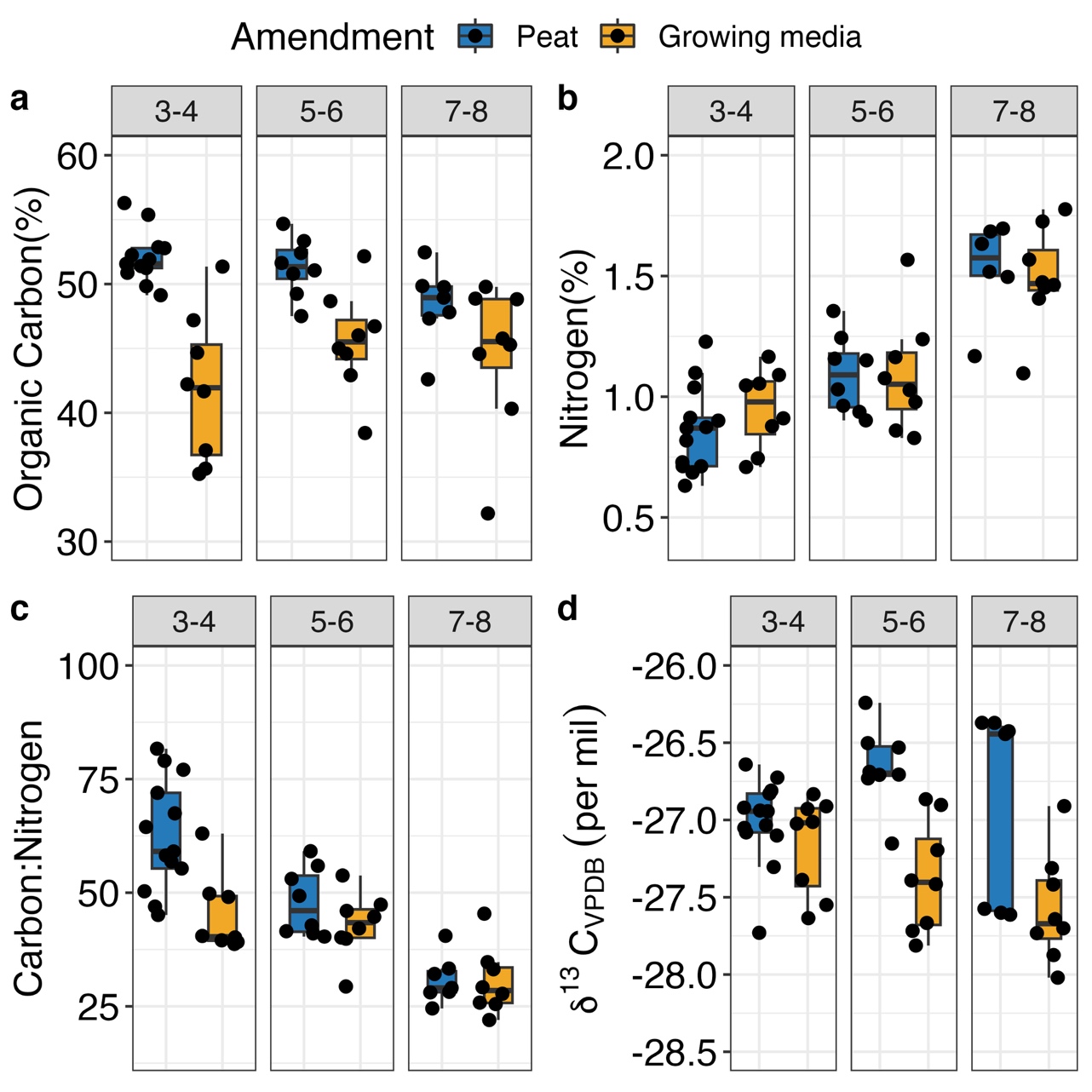


A

A

B

B

A

A

D

B

BDE

CE

A

BC

A

AC

BC

A

B

A

B

AD

BD

A

A

C

*Figure S2: a) Organic carbon (mass-%), b) nitrogen (mass-%), c) C:N ratio (g g^-1^) and* $\delta$*^13^C values for peat and growing media across different von Post scale.*

*
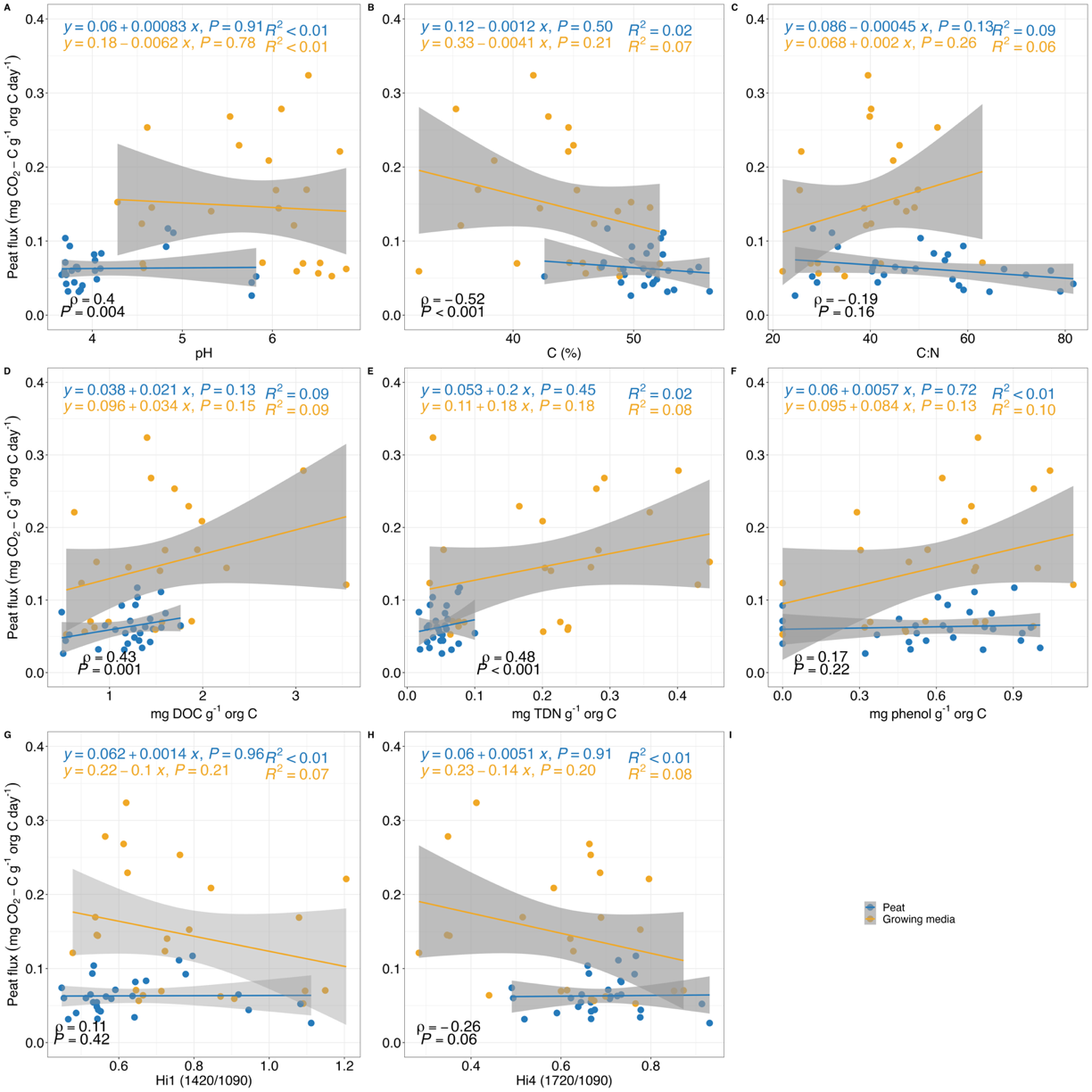
*

*Figure S3: Relations between peat-borne CO_2_-C emission with a) pH b) C c)C:N d) DOC e) TDN f) phenolic concentration g) Humification index (1420/1090) and h) humification index (1720/1090). Equation, p-value and R^2^ values represent the linear relationship between two variables for subset of peat and growing media.* $\rho$ *and p-value at the bottom left of the graph represents spearman correlation and associated p-value for the whole dataset. The shaded area around the lines represents 95% confidence interval.*

Table S2: Correlation between variables for growing media samples only. Symbols of *, ** and *** represent significance level at 0.1, 0.05 and 0.01 respectively.

|  | pH | LOI | Bulk density | DOC | TDN | DOC: TDN | Carbon | Nitrogen | C: N | Hi1 | Hi2 | Hi3 | Hi4 | Phenolic conc. | δ^13^ C- Peat |
| --- | --- | --- | --- | --- | --- | --- | --- | --- | --- | --- | --- | --- | --- | --- | --- |
| LOI | -.40* |  |  |  |  |  |  |  |  |  |  |  |  |  |  |
| Bulk density | .57*** | 0 |  |  |  |  |  |  |  |  |  |  |  |  |  |
| DOC | -0.27 | -0.3 | -.58*** |  |  |  |  |  |  |  |  |  |  |  |  |
| TDN | 0 | -0.25 | 0.14 | 0.19 |  |  |  |  |  |  |  |  |  |  |  |
| DOC: TDN | -0.19 | 0.04 | -.51** | .38* | -.77*** |  |  |  |  |  |  |  |  |  |  |
| Carbon | -0.33 | .47** | 0.05 | -.51** | -0.33 | -0.05 |  |  |  |  |  |  |  |  |  |
| Nitrogen | .38* | 0.22 | .42** | -.68*** | 0.05 | -.45** | 0.32 |  |  |  |  |  |  |  |  |
| C: N | -.62*** | 0.09 | -0.32 | .44** | -0.13 | .37* | 0 | -.79*** |  |  |  |  |  |  |  |
| Hi1 | 0.27 | 0.3 | .63*** | -.59*** | 0.06 | -.38* | 0.21 | .66*** | -.43** |  |  |  |  |  |  |
| Hi2 | 0.25 | 0.3 | .60*** | -.63*** | 0.01 | -.35* | 0.25 | .69*** | -.45** | .98*** |  |  |  |  |  |
| Hi3 | 0.26 | 0.29 | .61*** | -.60*** | 0.06 | -.39* | 0.19 | .64*** | -.41** | .98*** | .98*** |  |  |  |  |
| Hi4 | 0.31 | 0.32 | .70*** | -.68*** | 0.06 | -.46** | 0.25 | .64*** | -.43** | .83*** | .81*** | .87*** |  |  |  |
| Phenolic conc. | -0.25 | -0.19 | -.37* | .66*** | 0.15 | 0.33 | -0.23 | -.51** | 0.32 | -.59*** | -.65*** | -.64*** | -.65*** |  |  |
| δ^13^ C- Peat | -0.26 | 0.07 | -.75*** | 0.21 | -0.34 | .53*** | -0.03 | -0.18 | 0.16 | -0.31 | -0.27 | -0.3 | -.49** | 0.1 |  |
| CO_2_ emission | -0.2 | -0.14 | -0.28 | .35* | 0.26 | 0.08 | -.37* | -.48** | 0.35* | -0.32 | -0.34* | -0.29 | -0.26 | 0.32 | 0.21 |

Table S3: Correlation between variables for peat samples only. Symbols of *, ** and *** represent significance level at 0.1, 0.05 and 0.01 respectively.

|  | pH | LOI | Bulk density | DOC | TDN | DOC: TDN | Carbon | Nitrogen | C: N | Hi1 | Hi2 | Hi3 | Hi4 | Phenolic conc. | δ^13^ C- Peat |
| --- | --- | --- | --- | --- | --- | --- | --- | --- | --- | --- | --- | --- | --- | --- | --- |
| LOI | -.64*** |  |  |  |  |  |  |  |  |  |  |  |  |  |  |
| Bulk density | 0.19 | -.46** |  |  |  |  |  |  |  |  |  |  |  |  |  |
| DOC | -0.15 | -0.07 | -0.04 |  |  |  |  |  |  |  |  |  |  |  |  |
| TDN | .34* | -0.1 | 0 | 0.1 |  |  |  |  |  |  |  |  |  |  |  |
| DOC: TDN | -.47** | 0.11 | 0.04 | .41** | -.80*** |  |  |  |  |  |  |  |  |  |  |
| Carbon | -0.29 | .38** | -0.14 | 0 | -.33* | .37* |  |  |  |  |  |  |  |  |  |
| Nitrogen | .40** | -.42** | 0.19 | -0.3 | 0.25 | -.52*** | -.35* |  |  |  |  |  |  |  |  |
| C: N | -.41** | .42** | -0.14 | 0.28 | -.33* | .58*** | .47** | -.98*** |  |  |  |  |  |  |  |
| Hi1 | .52*** | -.74*** | .52*** | -0.16 | 0.04 | -0.23 | -.45** | .65*** | -.63*** |  |  |  |  |  |  |
| Hi2 | .55*** | -.72*** | .44** | -0.17 | 0.04 | -0.25 | -.47** | .78*** | -.77*** | .96*** |  |  |  |  |  |
| Hi3 | .53*** | -.73*** | .48*** | -0.17 | 0.05 | -0.25 | -.47** | .69*** | -.69*** | .99*** | .98*** |  |  |  |  |
| Hi4 | .49*** | -.71*** | .56*** | -0.18 | -0.02 | -0.19 | -.37* | .60*** | -.57*** | .97*** | .93*** | .95*** |  |  |  |
| Phenolic conc. | -0.12 | 0.16 | 0.05 | 0.31 | -0.18 | 0.29 | 0.19 | 0.04 | 0.05 | 0.01 | 0.09 | 0.06 | 0.08 |  |  |
| δ^13^ C- Peat | -0.07 | 0.09 | -0.14 | 0.31 | .37* | -0.14 | -0.18 | 0.21 | -0.25 | 0.08 | 0.1 | 0.12 | -0.01 | 0.09 |  |
| CO_2_ emission | 0.05 | -0.05 | 0.19 | 0.31 | 0.08 | 0.11 | -0.15 | 0.24 | -0.26 | 0.09 | 0.19 | 0.09 | 0.06 | 0.09 | .39** |
